# Multi-Objective Optimization of Machining Parameters for Drilling LM5/ZrO_2_ Composites Using Grey Relational Analysis

**DOI:** 10.3390/ma16103615

**Published:** 2023-05-09

**Authors:** Sunder Jebarose Juliyana, Jayavelu Udaya Prakash, Robert Čep, Krishnasamy Karthik

**Affiliations:** 1 Department of Mechanical Engineering, Vel Tech Rangarajan Dr. Sagunthala R&D Institute of Science and Technology, Chennai 600062, India; jebarose@veltech.edu.in (S.J.J.); udayaprakashj@gmail.com (J.U.P.); 2 Department of Machining, Assembly and Engineering Metrology, Faculty of Mechanical Engineering, VSB-Technical University of Ostrava, 17. Listopadu 2172/15, 708 00 Ostrava, Czech Republic; robert.cep@vsb.cz

**Keywords:** composites, drilling, grey relational analysis, design of experiments, ANOVA

## Abstract

In today’s world, engineering materials have changed dramatically. Traditional materials are failing to satisfy the demands of present applications, so several composites are being used to address these issues. Drilling is the most vital manufacturing process in most applications, and the drilled holes serve as maximum stress areas that need to be treated with extreme caution. The issue of selecting optimal parameters for drilling novel composite materials has fascinated researchers and professional engineers for a long time. In this work, LM5/ZrO_2_ composites are manufactured by stir casting using 3, 6, and 9 wt% zirconium dioxide (ZrO_2_) as reinforcement and LM5 aluminium alloy as matrix. Fabricated composites were drilled using the L_27_ OA to determine the optimum machining parameters by varying the input parameters. The purpose of this research is to find the optimal cutting parameters while simultaneously addressing the thrust force (TF), surface roughness (SR), and burr height (BH) of drilled holes for the novel composite LM5/ZrO_2_ using grey relational analysis (GRA). The significance of machining variables on the standard characteristics of the drilling as well as the contribution of machining parameters were found using GRA. However, to obtain the optimum values, a confirmation experiment was conducted as a last step. The experimental results and GRA reveal that a feed rate (F) of 50 m/s, a spindle speed (S) of 3000 rpm, Carbide drill material, and 6% reinforcement are the optimum process parameters for accomplishing maximum grey relational grade (GRG). Analysis of variance (ANOVA) reveals that drill material (29.08%) has the highest influence on GRG, followed by feed rate (24.24%) and spindle speed (19.52%). The interaction of feed rate and drill material has a minor impact on GRG; the variable reinforcement percentage and its interactions with all other variables were pooled up to the error term. The predicted GRG is 0.824, and the experimental value is 0.856. The predicted and experimental values match each other well. The error is 3.7%, which is very minimal. Mathematical models were also developed for all responses based on the drill bits used.

## 1. Introduction

Aluminium matrix composites (AMCs) have gained the attention of many scientists because the Al alloy overcomes the shortcomings of ferrous metals and provides the best specified performance parameters. AMCs are popular materials for meeting all of the rigorous demands in technical applications that require properties such as low weight, high stiffness, and medium strength [1,2,3]. AMCs combine the metal properties of matrix alloys with ceramic reinforcements to produce complex service temperature capabilities, increased strength, and compression resistance [4,5,6]. Noorul Haq et al. (2008) proposed a comprehensive two-dimensional array with the GRA method for optimising process parameters for drilling Al/SiC composites [7]. Ponnuvel and Moorthy (2014) studied the impact of drilling parameters on hybrid polymer composites (MWCNTs) [8]. Optimal cutting parameters were discovered using GRA while simultaneously addressing the TF, SR, and BH of drilled holes. The grey-fuzzy method was applied by Rajmohan et al. (2013) to find the best machining parameters for drilling composites of hybrid aluminium metal matrix. In the experiments, a three-level OA L_27_ is used [9]. Emin Salura et al. (2019) used a hot press to create MMC, and the effects of the output variables on the TF and SR of composites were studied using ANOVA [10]. The effects of production variables were investigated and visualised. It is possible to determine the ideal value for each output factor using the S/N ratio approach. The findings demonstrated that the additional phase material proportion was the primary determinant of the SR of the MMCs for both feed rates. As the feed rate increased during machining, the TF and SR values increased, according to the literature. However, when the feed rate increased, the TF and SR values in this MMC system decreased, making this study more novel. Palanikumar et al. (2012) employed GRA to optimise the drilling parameters for GFRP composite drilling based on SR and TF. The Taguchi L_9_ 3-level OA is employed in the experiment. Using the GRG acquired from the GRA, they established the pre-eminent parameters for multi-performance features [11]. Davim (2001) examined the consequences of process parameters on the surface quality of turned components. Experiments based on Taguchi’s methodologies were planned and carried out on controlled machining of workpieces with predetermined cutting conditions. Those associations were discovered using multiple linear regressions. Finally, they used confirmation tests to compare the theoretical outcomes with the expected results from the correlations [12]. Samy and Kumaran (2017) concluded the effects of cutting factors on temperature, TF, and SR on AA6351/B_4_C composite materials during drilling operations. Completely different angles of titanium nitride-coated carbide drill bits, such as 90°, 118°, and 135°, were employed. Lower spindle speeds and feed rates are used to achieve the much lower temperature. Unsuitable point angles, on the other hand, cause tool wear and increase surface roughness. Changing the machining factors also affects the production of thrust force. The thrust force is minor when drilling with a tool with a 135° point angle, but the delamination is greater [13].

According to Junfeng Xiang et al. (2017), SiCp/Al matrix composite materials have numerous remarkable physical and mechanical properties. Their goal is to investigate the mechanisms that influence the macro-scale wear of diamond tools when drilling SiCp/Al composite materials. Aside from that, drilling forces and hole reliability were used to assess the machinability of the SiCp/Al 6063 composite material. The findings imply that mechanically generated abrasive wear and thermodynamically allowed chemical graphification are important and likely wear mechanisms in SiCp/Al6063 drilling. Diamond-coated carbide drills are chosen for composite material because of their longlife, low wear rate, and ability to generate sufficient machined efficiency with a growing number of holes [14]. Jayaganth et al. (2018) used Taguchi’s L_9_ array to conduct drilling tests with various cutting speeds, feeds, and cutting fluids. Surface roughness values decrease, but machining time increases as cutting speed and feed rate increase. Optimal process parameters have been designed and confirmed to improve machinability. At higher speeds and feeds, the coconut oil medium provided superior machinability [15]. Mahamani (2014) explored the effects of feed, speed, point angle, and drill bit diameter on surface roughness (SR) in the drilling of AA2219/TiB_2_/ZrB_2_ hybrid composites. Investigational analysis explores the influence of one process variable while keeping the other variables constant [16]. Ekici et al. (2017) assessed the effects of process parameters on thrust force (TF), SR, dimensional accuracy, and burr height (BH) when drilling Al/10B_4_C and Al/10B_4_C/5Gr composites using carbide twist drills at various levels of speeds and feed rates under dry cutting conditions. ANOVA was used to evaluate the percentage contribution of process variables to product quality. Finally, they developed statistical equations to evaluate quality attributes. Al/10B_4_C/5Gr, a second-phase material with 5% graphite, decreased the TF and BH of the composites, enhancing the surface’s quality [17].

Ravindranath et al. (2017) examined the consequences of process parameters during the drilling of Al2219/8% boron carbide (B_4_C) composite and hybrid composite Al2219/8% B_4_C/3% Gr. The experiment was carried out at various speeds and feeds; the impact of the TF and SR was examined, and the findings show that when the FR rose, the TF and SR increased. Because graphite has good lubricating characteristics, hybrid composites have lower thrust and a larger surface roughness [18]. In drilling SA182 work material, Sunil Ankalagi et al. (2017) studied the outcome of drilling variables such as F, S, and drill point angle on TF, SR, and circularity error. To explain the behaviour of machinability and hole quality, the investigations were designed using an orthogonal array (OA) and response surface methodology (RSM). The feasibility of the models for the indicated responses was validated using an ANOVA. All the responses were found to decrease with increasing spindle speed, while the circularity error decreased with a high cutting speed and feed, according to the response surface analysis. Circularity error and surface roughness were reduced when the point angle was increased [19]. This research work’s objective is to conduct multi-objective optimisation of drilling process parameters for achieving minimum TF, SR, and BH of LM5/ZrO_2_ composite and to investigate the effect of process parameters on TF, BH, and SR.

## 2. Materials and Methods

### 2.1. Materials

Due to its widespread availability, zirconia (ZrO_2_) has been chosen as the reinforcement material and LM5 aluminium alloy as the matrix. The alloy is used for devices used in the production of foodstuffs, culinary utensils, and chemical industries, as well as for the moulding of fine polish areas where quite strong corrosion resistance from saltwater or marine atmospheres is desired. They are very well known for their aesthetic casts as well as casts utilised in applications for building and decorative maritime fittings, food handling dairy equipment, and chemical and maritime plumbing fittings [20]. LM5 is examined for its chemical composition by means of optical emission spectrometry, and it is presented in Table 1.

Zirconium dioxide (ZrO_2_), a crystalline oxide of zirconium (zirconia), which is white in colour, is broadly considered to be a ceramic element. Making zirconia includes the gathering and removal of laid-off ingredients and scum. Mining zirconia has many routes, including plasma disassociation, chlorine and alkali oxide disintegration, and lime amalgamation. Similar to many ceramics, zirconium oxide is a substrate with a high tolerance to crack propagation. ZrO_2_ ceramics are thermally established, and often they are the material for linking ceramics and steel. Little thermal conductivity and great strength are another awful combination of properties.

### 2.2. Manufacturing of LM5/ZrO_2_ Composites

To fabricate the composite, a closed furnace, which is of the C-type, is used with the stir casting set-up. The stirrer incorporates a chuck for convenient shaft interchangeability. It has a four-bladed fan impeller that is made of high-chromium steel. Initially, small ingots of LM5 alloy were heated to around 850 °C in the crucible until the entire alloy was melted. To eradicate dampness in the reinforcement, the ZrO_2_ powder is dried for 20 min at 200 °C using a muffle furnace. The stirrer was progressively inserted into the melt, creating a vortex in the molten metal. The warmed ZrO_2_, with an average particle size of 60 to 80 µm, was then carefully and slowly mixed into the liquid metal at a consistent rate while upholding the speed of the stirrer at 600 rpm. Even after particle feeding, the stirring continued for another 7 min. To reduce porosity, argon gas was added to the slurry for three minutes before pouring it into the mould. The pouring temperature was fixed at 750 °C. To achieve uniform solidification, the mould was warmed to 650 °C for 30 min before pouring the slurry into it. This method was used to create three distinct sets of unique composites constructed of LM5 reinforced with 3, 6, and 9 weight percents of ZrO_2_ particles [21].

### 2.3. Microstructure of Fabricated Composites Using Optical Microscopy

Metallographic examinations provide a significant investigative tool and effective quality control. Samples were collected from every composite, and each surface was finely polished to achieve a mirror-like sheen. The primary goal of a microstructural analysis is to validate the uniform dispersion of a matrix’s reinforcement particles.

**Figure 1:**
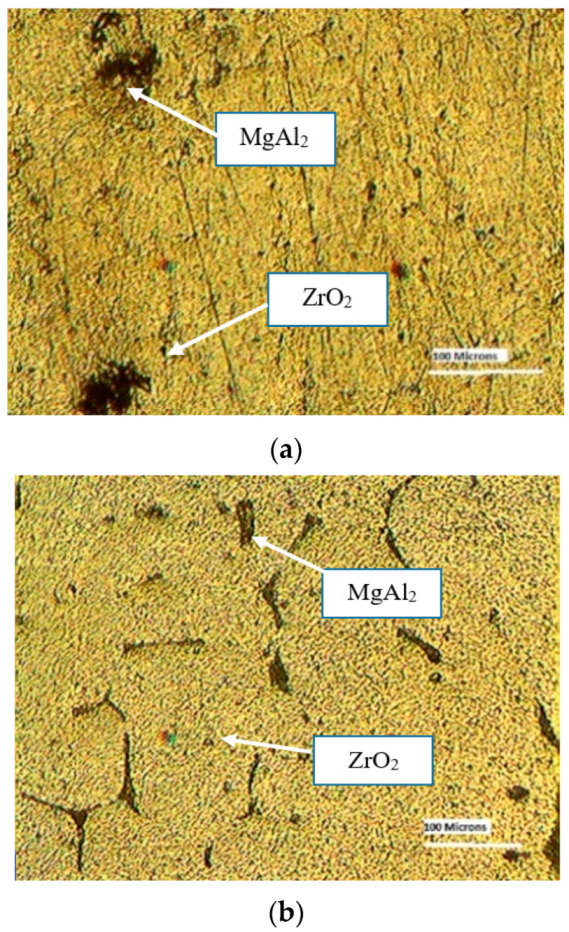

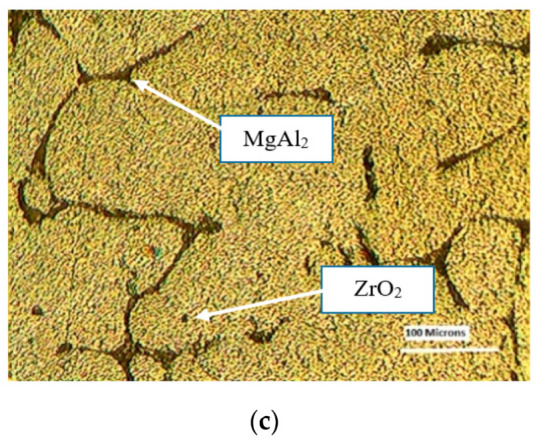
Microstructures of fabricated composites. (**a**) LM5 + 3%ZrO_2_; (**b**) LM5 + 6%ZrO_2_; (**c**) LM5 + 9%ZrO_2_.

The optical microscope was used to investigate the composite specimens. The homogenous spreading of reinforcement particles in the matrix is shown in the optical photomicrographs (Figure 1). The dispersion of composite ZrO_2_ particles is seen in the microstructure of metal matrix composites containing 3% and 6% ZrO_2_. The primary aluminium grains contain the particles. MgAl_2_ eutectic particles that did not dissolve after solidification are precipitated at the grain boundaries. The magnification is 200×. The main aluminium phase includes grains that are 40 to 60 microns in size. In the 9% ZrO_2_ composite, particle dispersal is detected and exists as lateral bunches on the grain boundaries, although the micrograph only displays the resolved composite particles.

### 2.4. Drilling of LM5/ZrO_2_Composites

**Figure 2:**
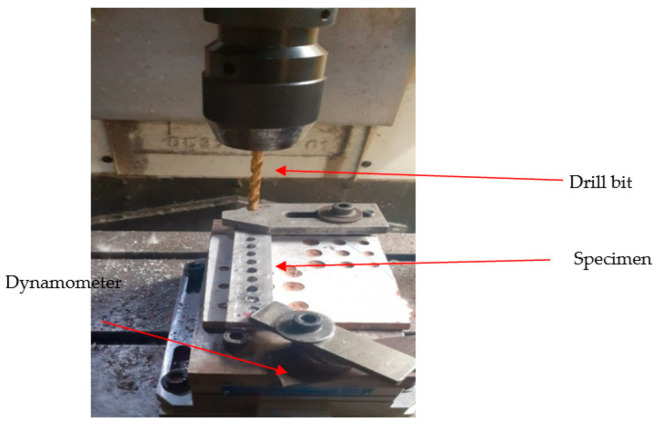
Experimental set-up with a dynamometer.

**Figure 3:**
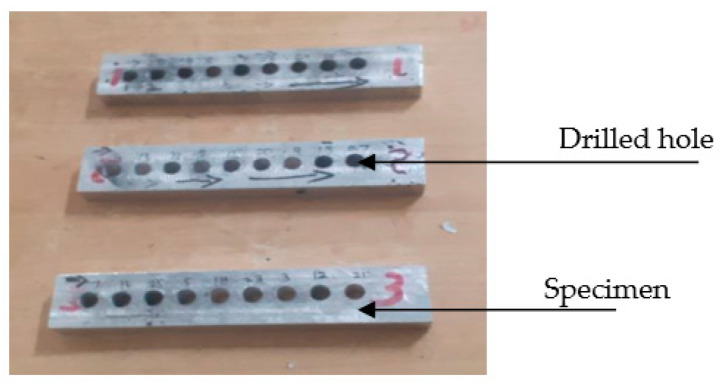
Drilled holes.

The Gaurav-BMV 35 T12 (Model) Vertical Machining Centre (VMC), equipped with a Kistler peizo-electric dynamometer shown in Figure 2, is used for drilling holes in composite materials. In Figure 3, specimen 1 represents LM5 + 3%ZrO_2,_ specimen 2 represents LM5 + 6%ZrO_2_ and specimen 3 represents LM5 + 9%ZrO_2._ A computer-controlled data collection tool captures and stores the results of experiments. The reliability of the drilled hole is the primary concern during drilling. A piezo-electric dynamometer was used to measure the TF generated during drilling, which is the main determinant of the hole’s quality. BH was found using the Vision Measuring System (VMS), and SR was determined using the surface roughness tester—surf corder [22]. Cutting tools used in this research work were made of three different materials: HSS, carbide, and titanium nitride (TiN)-coated carbide. For all three drills, the diameter is 6 mm, the point angle is 118°, and the helix angle is 30°. The experiment’s four primary process parameters were picked. The three levels of drilling variables are presented in Table 2.

### 2.5. Grey Relational Analysis

Multi-performance characteristics are tough to optimise in complex processes; hence, GRA is largely employed to address such a tough problem. The advantages of grey system theory have been confirmed in coping with the challenges of incomplete, partial, and unclear data. The terms black, white, and grey are defined differently in grey relational analysis. Black denotes a system with no information, white denotes a system with exact information, and grey represents the information between black and white. This technique addresses the issue of improving the response features of current machining systems [23,24].

In grey relational analysis, the first step is data pre-processing, where the TF, SR, and BH experimental data are standardised to be in the range of zero to one. Data pre-processing is typically necessary because the range and unit of one data set differ from the others. The method of converting a sequence into one that is comparable to the original is called data pre-processing. Depending on a data series’ characteristics, there are a number of data pre-processing methods accessible for GRA. The “higher-the-better” characteristic applies if the target value is infinite. The sequence can be normalised as shown in Equation (1):

(1) $$x_{i}^{*}(k)=\frac{x_{i}^{\left(0\right)}(k)-\min x_{i}^{\left(0\right)}(k)}{\max x_{i}^{\left(0\right)}(k)-\min x_{i}^{\left(0\right)}(k)}$$

When the original sequence has the“lower-the-better” characteristic, it shouldbe normalised as shown in Equation (2):

(2) $$x_{i}^{*}(k)=\frac{\max x_{i}^{\left(0\right)}(k)-x_{i}^{\left(0\right)}(k)}{\max x_{i}^{\left(0\right)}(k)-\min x_{i}^{\left(0\right)}(k)}$$

where *i* = 1, 2, …, m and k = 1, 2, …, n signify the original reference sequence and pre-processed data, respectively. $x_{i}^{*}$(k) denotes the normalised value, $x_{i}^{\left(0\right)}$ represents the intended sequence, min $x_{i}^{\left(0\right)}(k)$ denotes the sequence’s minimum value, and max $x_{i}^{\left(0\right)}(k)$ represents the sequence’s maximum value. The total number of observations is n, whereas there are m experiments.

### 2.6. Grey Relational Coefficient (GRC)

A metric used in GRA to judge the applicability of two systems or sequences is the GRC. Equation (3) illustrates the GRC, which is used in GRA to show how closely related the sequences of *x*_0_(*k*) and *x_i_*(*k*) are to one another.

(3) $$\gamma x_{0}(k),x_{i}^{*}=\frac{\Delta_{min}+\zeta \Delta_{max}}{\Delta_{0i}(k)+\zeta \Delta_{max}}$$

where *Δ*_0*i*_(*k*) is also recognised as the deviation sequence and replicates the change between *x*_0_(*k*) and $x_{i}^{*}$(*k*).

*∆*_0_*_i_*(*k*) *= x*_0_(*k*) − $x_{i}^{*}$(*k*),

*∆_min_* is the smallest value of *∆_0i_*(*k*),

*∆_max_* is the largest value of *∆_0i_*(*k*), and 

ζ is the distinguishing coefficient.

In most cases, the ζ value is smaller and the distinct ability is larger, so ζ = 0.5 is employed.

### 2.7. Grey Relational Grade (GRG)

By calculating the GRC, the GRG is usually calculated using the average value of the GRC. GRG is used to evaluate multi-response characteristics. Equation (4) shows how it is expressed:

(4) $$\tau_{i}=\frac{1}{n}\sum_{i=1}^{n}\left(\gamma (x_{0}(k),x_{i}^{*}(k)\right)$$

where *n* is the number of process responses and $\tau_{i}$ is the GRG. The greater GRG shows that the associated experimental result is closer to the ideal normalised value.

### 2.8. Predicting the Optimal Responses

The optimal levels of each factor are used to predict the best answers using the best levels of each element in the Taguchi design of experiments, followed by confirmation trials. Depending on whether the experiment’s objective is to minimise or maximise the response, the best condition is selected [25,26]. When there are three levels, 1, 2, and 3, with level 1 being the ideal situation, and there are three components, A, B, and C, Equation (5) provides the predicted optimal response.

(5) $$\mu_{pred}=(\overset{-}{A_{1}}+\overset{-}{B_{1}}+\overset{-}{C_{1}})-2\overset{-}{Y}$$

where

$\overset{-}{Y}$ is the overall mean response, and 

$\overset{-}{A_{1}},\overset{-}{B_{1}},\overset{-}{C_{1}}$ are the average responses at level 1 of these factors.

## 3. Results and Discussions

### 3.1. Experimental Results

Utilising GRA, multi-objective optimisation was performed with the intention of producing the minimum TF, SR, and BH simultaneously. Table 3 lists the trial outcomes and their GRC, GRG, and ranks.

### 3.2. Analysis and Discussion of Results

**Figure 4:**
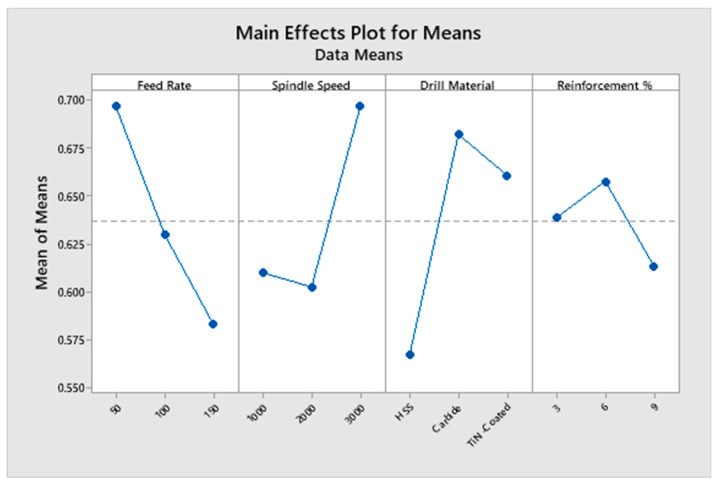
Response graphs for GRG.

The experiments are ranked according to the GRG values. Figure 4 evidently displays that GRG is high for the first level of F, the third level of SS, and the second levels of D and R% A_1_B_3_C_2_D_2_.

The mean response values for each factor level are shown in Table 4. The rank shows that the D has more significance on the GRG, followed by the F, S, and R%. The actual experimental strategy was evaluated with a 95% level of confidence. The findings of the GRG ANOVA are listed in Table 5. The obtained R^2^ value for GRG is 80.47%. For F, SS, and D, the *p*-value is less than 0.05, indicating that they have a significant impact on the GRG.

The tabulated F-value is F_0.05, 2, 16_ = 3.63. It is obvious from Table 5 that the F-tested values for F, S, and D are greater than the F-tabled value, and hence they have a significant impact on GRG.The F test value of R% and interactions of F with other variables are less than the tabulated value. It is noted that D (29.08%) has the maximum influence on the GRG, followed by F (24.24%) and S (19.52%). The response variable R% and the interactions of F with every other variable were pooled up to the error term as it does not have a major impact on GRG [27,28].

### 3.3. Confirmation Experiments

The results of the confirmation experiments show that the predicted GRG is 0.824 and the experimental value is 0.856. A good agreement is found between the predicted and investigational values, and the error is 3.7%.

### 3.4. Influence of Input Variables on GRG

**Figure 5:**
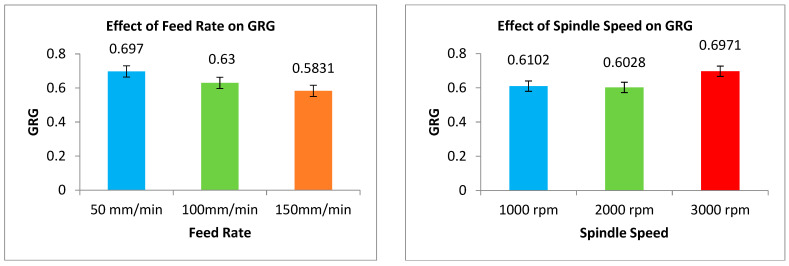

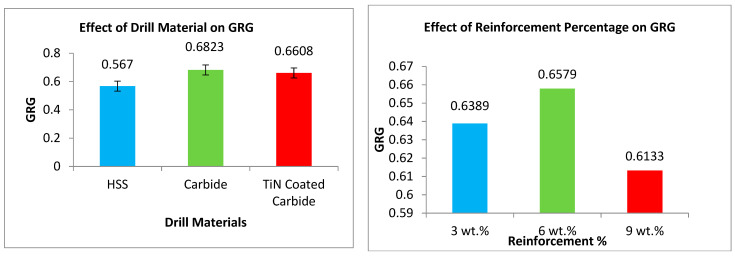
Effect of drilling process parameters on GRG.

The TF, SR, and BH responses were included in the GRG as a high-quality depiction of all the responses. Figure 5 displays the impact of the factors on the GRG. The GRG response graph’s greatest value indicates that drilling factors had a higher influence on machinability attributes [29,30]. The best process parameters for drilling were F = 50 mm/min, S = 3000 rpm, D = Carbide, and R = 6 wt%, which led to the maximal value of the GRG. The maximum GRG was found at the lowest F and highest S, suggesting that the response TF, SR, and BH were at their lowest levels at the lowest F and highest S. This is due to a lower TF resulting from a decrease in the amount of friction between the drill bit and the specimen [31,32]. A lower F indicates a lower drilling temperature, which enhances the quality of the surface. A lower F was found to result in a lower TF, which provided a satisfactory surface finish at a smaller F. The workpiece softens and penetrates smoothly as a result of the quick heat rise caused by friction at higher spindle speeds, which results in a smaller TF. Better GRG values are produced by increasing spindle speed because shorter cutting times lead to less thrust force, less workpiece deformation, and shorter overall times. Compared to the other two weight percents, the SR value for 6% ZrO_2_ was lower. With the addition of ZrO_2_, the TF value in the LM5/ZrO_2_ composite material increased. Because ZrO_2_ is the hardest material, raising the ZrO_2_% causes the composite’s hardness to increase as well, causing the TF to increase. When the weight percentage of the reinforcement rises, the SR decreases. The cutting force and BH increase as the F rises. The BH is the highest for high feed rates. For LM5/ZrO_2_ composites, the BH falls as the reinforcement percentage increases [33,34]. The GRG value depends on the response TF, SR, and BH since it is based on the average of the GRC of the TF, the GRC of the SR, and the GRC of the BH of each experiment [35].

### 3.5. Mathematical Models

Mathematical models of LM5/ZrO_2_composites for thrust force (TF), surface roughness (SR), and burr height (BH) for an HSS drill are presented in Equations (6)–(8). Similarly, the equations developed for carbide and TiN-coated drills are presented in Equations (9)–(11) and (12)–(14), respectively.

For an HSS drill:

TF = 142.0 + 0.892 Feed Rate − 0.05678 Spindle Speed + 4.13 Reinforcement Percentage(6)

SR = 4.089 + 0.03031 Feed Rate − 0.000758 Spindle Speed − 0.0274 Reinforcement Percentage(7)

BH = 0.0556 − 0.000018 Feed Rate − 0.000001 Spindle Speed − 0.00017 Reinforcement Percentage(8)

For a carbide drill:

TF = 137.7 + 0.892 Feed Rate − 0.05678 Spindle Speed + 4.13 Reinforcement Percentage(9)

SR = 3.666 + 0.03031 Feed Rate − 0.000758 Spindle Speed − 0.0274 Reinforcement Percentage(10)

BH = 0.0354 − 0.000018 Feed Rate − 0.000001 Spindle Speed − 0.00017 Reinforcement Percentage(11)

For a TiN-coated carbide drill:

TF = 132.3 + 0.892 Feed Rate − 0.05678 Spindle Speed + 4.13 Reinforcement Percentage(12)

SR = 3.380 + 0.03031 Feed Rate − 0.000758 Spindle Speed − 0.0274 Reinforcement Percentage(13)

BH = 0.0354 − 0.000018 Feed Rate − 0.000001 Spindle Speed − 0.00017 Reinforcement Percentage(14)

## 4. Conclusions

Composites at three different weight percentages were manufactured using the low-cost stir casting method. The homogeneous distribution of the reinforcement material (ZrO_2_) in the matrix was confirmed using optical micrographs.

On LM5/ZrO_2_ composites, drilling experiments were carried out using Taguchi’s DoE and analysed using a grey relational analysis. The influence of drilling variables on LM5/ZrO_2_ composites led to the following conclusions: The TF, SR, and BH values decreased with a decrease in the feed rate for all the specimens. The most statistically noteworthy parameter on GRG is D (29.08%), followed by F (24.24%) and S (19.52%). The predicted GRG is 0.824, the experimental value is 0.856, and the error is 3.7%. The margin of error for the responses is minimal, according to confirmation studies. A good agreement is found between the predicted and experimental values.

## Figures and Tables

**Table 1:** Chemical composition of aluminium alloy (LM5).

Cu	Mg	Si	Mn	Fe	Pb	Zn	Al
0.032	3.299	0.212	0.022	0.268	0.02	0.01	Balance

**Table 2:** Drilling process parameters and their levels.

Factor	Level 1	Level 2	Level 3
F (mm/min)	50	100	150
S (rpm)	1000	2000	3000
D	HSS	Carbide	TiN Coated
R (%)	3	6	9

**Table 3:** Results of a grey relational analysis (LM5/ZrO_2_).

Sl No	Feed (mm/min)	Speed (rpm)	Drill Material	Reinforcement (wt %)	GRC of TF	GRC of SR	GRC of BH	GRG	Rank
1	50	1000	HSS	3	0.655	0.654	0.678	0.662	11
2	50	1000	Carbide	6	0.615	0.744	0.695	0.685	10
3	50	1000	TiN Coated	9	0.612	0.499	0.781	0.631	13
4	50	2000	HSS	6	0.7	0.434	0.648	0.594	17
5	50	2000	Carbide	9	0.84	0.744	0.496	0.693	9
6	50	2000	TiN Coated	3	0.904	0.433	0.76	0.699	8
7	50	3000	HSS	9	0.716	0.418	0.695	0.610	16
8	50	3000	Carbide	3	0.969	0.702	0.678	0.783	2
9	50	3000	TiN Coated	6	1	0.747	1	0.916	1
10	100	1000	HSS	3	0.481	0.436	0.678	0.532	24
11	100	1000	Carbide	6	0.421	1	0.864	0.762	3
12	100	1000	TiN Coated	9	0.454	0.583	0.695	0.577	19
13	100	2000	HSS	6	0.704	0.462	0.333	0.500	27
14	100	2000	Carbide	9	0.625	0.758	0.741	0.708	5
15	100	2000	TiN Coated	3	0.7	0.392	0.509	0.534	23
16	100	3000	HSS	9	0.652	0.468	0.760	0.627	14
17	100	3000	Carbide	3	0.846	0.581	0.678	0.702	7
18	100	3000	TiN Coated	6	0.762	0.487	0.935	0.728	4
19	150	1000	HSS	3	0.358	0.595	0.581	0.511	26
20	150	1000	Carbide	6	0.348	0.414	0.891	0.551	20
21	150	1000	TiN Coated	9	0.333	0.559	0.85	0.581	18
22	150	2000	HSS	6	0.53	0.411	0.634	0.525	25
23	150	2000	Carbide	9	0.497	0.522	0.634	0.551	21
24	150	2000	TiN Coated	3	0.577	0.516	0.77	0.621	15
25	150	3000	HSS	9	0.673	0.333	0.62	0.542	22
26	150	3000	Carbide	3	0.747	0.522	0.85	0.706	6
27	150	3000	TiN Coated	6	0.704	0.581	0.695	0.66	12

**Table 4:** Response table for GRG (LM5/ZrO_2_).

Level	Feed Rate (F)	Spindle Speed (S)	Drill Material (D)	Reinforcement% (R)
1	0.6970	0.6102	0.5670	0.6389
2	0.6300	0.6028	0.6823	0.6579
3	0.5831	0.6971	0.6608	0.6133
Delta	0.1139	0.0943	0.1153	0.0446
Rank	2	3	1	4

**Table 5:** ANOVA for GRG (LM5/ZrO_2_).

Source of Variation	DOF	SS	MS	F	*p*	C%
Feed Rate	2	10.574	5.2871	9.93	0.002	24.24
Spindle Speed	2	8.516	4.2582	8.00	0.004	19.52
Drill Material	2	12.688	6.3442	11.92	0.001	29.08
Feed rate*Drill Material	4	3.326	0.8315	1.56	0.232	7.62
Pooled Error	16	8.519	0.5324			19.53
Total	26	43.624				100.00

## Data Availability

The data presented in this study are available through email upon request to the corresponding author.
